# Association between Maternal Zinc Status, Dietary Zinc Intake and Pregnancy Complications: A Systematic Review

**DOI:** 10.3390/nu8100641

**Published:** 2016-10-15

**Authors:** Rebecca L. Wilson, Jessica A. Grieger, Tina Bianco-Miotto, Claire T. Roberts

**Affiliations:** 1 Robinson Research Institute, University of Adelaide, Adelaide SA 5005, Australia; rebecca.l.wilson@adelaide.edu.au (R.L.W.); jessica.grieger@adelaide.edu.au (J.A.G.); tina.bianco@adelaide.edu.au (T.B.-M.); 2 Adelaide Medical School, University of Adelaide, Adelaide SA 5005, Australia; 3 Waite Research Institute, School of Agriculture, Food and Wine, University of Adelaide, Adelaide SA 5005, Australia

**Keywords:** zinc, pregnancy, pregnancy complications, dietary zinc intake, circulating zinc

## Abstract

Adequate zinc stores in the body are extremely important during periods of accelerated growth. However, zinc deficiency is common in developing countries and low maternal circulating zinc concentrations have previously been associated with pregnancy complications. We reviewed current literature assessing circulating zinc and dietary zinc intake during pregnancy and the associations with preeclampsia (PE); spontaneous preterm birth (sPTB); low birthweight (LBW); and gestational diabetes (GDM). Searches of MEDLINE; CINAHL and Scopus databases identified 639 articles and 64 studies were reviewed. In 10 out of 16 studies a difference was reported with respect to circulating zinc between women who gave birth to a LBW infant (≤2500 g) and those who gave birth to an infant of adequate weight (>2500 g), particularly in populations where inadequate zinc intake is prevalent. In 16 of our 33 studies an association was found between hypertensive disorders of pregnancy and circulating zinc; particularly in women with severe PE (blood pressure ≥160/110 mmHg). No association between maternal zinc status and sPTB or GDM was seen; however; direct comparisons between the studies was difficult. Furthermore; only a small number of studies were based on women from populations where there is a high risk of zinc deficiency. Therefore; the link between maternal zinc status and pregnancy success in these populations cannot be established. Future studies should focus on those vulnerable to zinc deficiency and include dietary zinc intake as a measure of zinc status.

## 1. Introduction

Adequate maternal nutrition, particularly before and during pregnancy, is imperative to the health of both the mother and child [1,2]. Poor nutrition in pregnancy may lead to inappropriate nutrient partitioning between the mother and fetus, which can be deleterious to the health of both [3]. Each year, 3.5 million deaths in women and children are attributed to undernutrition [4]. Zinc deficiency is predicted to be responsible for 1% of all deaths globally and 4.4% of deaths in children aged 6 months to 5 years [5]. The World Health Organization (WHO) prioritized minimizing zinc deficiency in developing nations as part of the Millennium Development Goal 1: to eradicate extreme poverty and hunger [6]. Therefore, understanding the effects of zinc deficiency on pregnancy and fetal growth is very important.

Zinc is an essential component of over 1000 proteins including antioxidant enzymes, metalloenzymes, zinc-binding factors and zinc transporters. These are required for a variety of biological processes including carbohydrate and protein metabolism, DNA and RNA synthesis, cellular replication and differentiation, and hormone regulation [7,8,9,10]. The importance of zinc to the growth of the fetus is demonstrated by the active transport of zinc across the placenta into the fetal circulation resulting in higher cord blood concentrations compared to those in the maternal circulation [11,12,13,14]. Rodent models of severe maternal zinc deficiency show increased rates of fetal loss and congenital malformations in the surviving fetuses [15] as well as reduced fetal growth [16,17,18], lower implantation rates and impaired placental growth [19], all highlighting the teratogenic effects of zinc deficiency in pregnancy.

Diet is the main factor that determines zinc status [20]. In the United States and Australia, an additional 2–4 mg zinc per day is recommended for pregnant women compared to non-pregnant women [21,22]. It is widely acknowledged that many pregnant women do not meet this recommendation [23,24,25], particularly in developing countries where diets are often plant-based. Grains and legumes contain a significant amount of phytic acid and phytate binding of zinc limits its absorption in the small intestine, contributing to zinc deficiency [22]. Estimates based on bioavailability of zinc, physiological requirements and predicted zinc absorption suggest the prevalence of zinc deficiency to range from 4% (European countries including the United Kingdom, Sweden, Germany and France) to 73% in Bangladesh, India and Nepal [26]. A more recent evaluation, based on similar estimates, also predicted inadequate zinc intakes in over 25% in populations in Southeast Asia and Africa [27].

A recent Cochrane review assessed the effects of zinc supplementation versus no supplementation (with or without placebo) on the success of pregnancy in 21 randomized controlled trials (RCTs) [28]. It was concluded that zinc supplementation reduced the risk of spontaneous preterm birth (sPTB) by 14% (RR: 0.86, 95% CI: 0.76–0.97; 16 RCTs) but there was no effect on other outcomes such as stillbirth/neonatal death, birthweight and pregnancy-induced hypertension [28]. However, this review did not include the effects of zinc supplementation on reducing the risk of gestational diabetes (GDM) and analysis of maternal circulating zinc concentrations provides evidence that low maternal zinc may be associated with GDM, as well as preeclampsia (PE), gestational hypertension (GH), sPTB and infant birthweight [24,29]. The association between serum zinc and PE has been reviewed recently [30] but there has been no extensive review that has assessed maternal zinc concentrations with respect to a range of pregnancy complications. Here, we review the current literature based on observational studies assessing the association between maternal zinc status and a number of pregnancy complications in order to determine whether maternal circulating zinc or dietary zinc intake are important factors associated with pregnancy outcome.

## 2. Materials and Methods

### 2.1. Eligibility Criteria

Studies included human prospective cohorts, case-control, longitudinal and cross-sectional studies assessing maternal circulating zinc concentrations and pregnancy complications including PE, eclampsia, GH, GDM, small for gestational age (SGA), intrauterine growth restriction (IUGR; ˂10th percentile), low-birthweight (LBW; ≤2500 g) and sPTB. Only studies that measured maternal circulating zinc during pregnancy or at delivery and/or dietary zinc intake at these times were included. Studies that assessed zinc concentrations in placenta, amniotic fluid, in offspring (post-natally), cord blood only and breast milk were excluded. There were no restrictions imposed on age of women included in the studies or on any other population characteristic such as race or body mass index (BMI). Given the heterogeneity of the observational strategies, a meta-analysis was not possible.

### 2.2. Information Sources and Search

The search strategy and procedure was guided by the PRISMA statement [31]. Potential studies were located through electronic databases (Ovid Medline (1946–present), CINAHL (1937–present) and Scopus (1995–present)), as well as manual searches of references in review articles and relevant articles known by the authors. Limits included full text articles written in English and published in academic journals. The last search was performed on 25 August 2016. Search terms and MeSH headings in the title, abstract, and index terms, were initially identified in Medline and subsequent key words were used for the remaining databases (Appendix A). Briefly, the search included the following: zinc; dietary zinc; zinc intake; plasma zinc; serum zinc; preeclampsia; eclampsia; gestational hypertension; gestational diabetes mellitus; fetal macrosomia; small for gestational age; intrauterine growth restriction; low birthweight; preterm birth.

### 2.3. Data Collection

An independent search of the literature was performed in April 2015 and again in August 2016. Titles and abstracts were examined independently by two of the authors who documented reasons for excluding full text articles. Any differences between the two reviewers were clarified; a third reviewer resolved any disagreements. If an article appeared in duplicate from two or three of the databases, only the search containing the most relevant and useful information was included. For each eligible study, the following data was extracted: author, year and country of publication; inclusion/exclusion criteria; sample size; zinc measure including sample type, collection time during pregnancy and method of analysis and pregnancy outcome. Most studies did not report on exclusion/inclusion criteria; these were therefore not included in the results table. Values determining zinc status were all converted to μg/L for easier comparisons between studies (Appendix B).

## 3. Results

**Figure 1:**
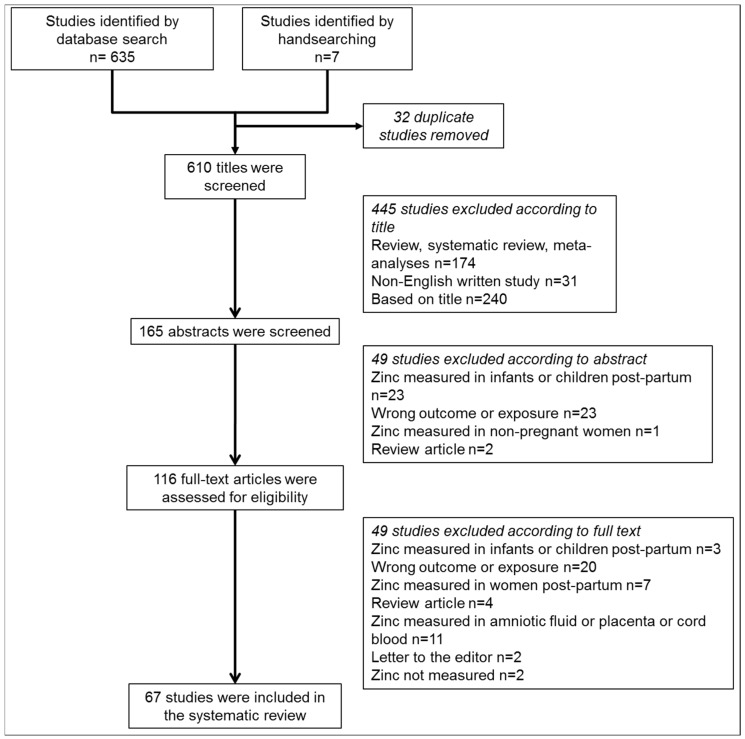
Flow diagram of the search strategy used in this review including the relevant number of papers at each point.

Figure 1 outlines the literature search and selection of studies. We identified 635 citations after searching Medline (OVID), CINAHL and Scopus databases. A further seven were added by the authors. After screening the title and abstract, 116 full text papers were read. Of these, 67 studies met the inclusion criteria, including 29 on SGA/LBW (Table 1), 34 on hypertensive disorders of pregnancy (Table 2), 11 on sPTB (Table 3) and 9 on GDM (Table 4). Eleven studies assessed multiple pregnancy outcomes and are included in the relevant pregnancy outcome tables. Table 5 summarizes all included studies and whether there was a positive, negative or no association between zinc status and the pregnancy complication. The included studies were tabulated based on those that measured dietary zinc intake, then those that measured serum/plasma zinc. Globally, the average percentage of people affected by inadequate zinc intake is estimated to be 17.3% [27]. As dietary consumption of zinc is most influential on zinc status, studies that measured circulating zinc were further categorized based on whether they sampled from countries where inadequate zinc intake has been predicted to affect <17% or ≥17% of the population. We did not limit the studies to a specific period during gestation when zinc was measured and this information was not provided in eight studies [32,33,34,35,36,37,38,39]. However, zinc concentrations decline across gestation due to a combination of factors including hemodilution and increased fetal demand [40,41] and this made direct comparison of the studies difficult.

### 3.1. Infant Birthweight

There were four studies that assessed dietary zinc intake and birthweight with three based on women from countries where the estimated prevalence of low dietary zinc intake is <17% (Table 1) [42,43,44,45]. Lower zinc intake was reported in women from the United Kingdom (UK) who gave birth to an SGA infant compared to those who gave birth to an appropriate-for-gestational-age (AGA) infant (SGA: mean (SEM) 11.3 (0.5) vs. AGA 13.0 (0.6) mg/day, *p* ˂ 0.05) [45]. This was similar to another study of Indian women that reported lower zinc intakes in women who delivered an infant weighing <2500 g compared to those who delivered an infant that was ≥2500 g [42]. Logistic regression analysis in one study from the United States reported daily zinc intake ˂6 mg/day to be associated with a 2-fold increase in the risk of delivering a LBW infant (aOR: 2.01, 95% CI: 1.11–3.66) [44] although dietary zinc intakes <median were not found to be associated with LBW in another study of American women (OR: 1.4, 95% CI: 0.9–2.1) [43]. While both studies used a 24 h recall questionnaire to determine zinc intakes, there were differences in ethnicity of the women studied as Neggers et al., [43] predominantly studied African-American women as opposed to Scholl et al. who studied Caucasian women [44].

Twelve studies were identified that measured maternal circulating zinc in countries where inadequate zinc intake is predicted to be <17%, and looked at the association with birthweight (Table 1) [38,46,47,48,49,50,51,52,53,54,55,56]. Only one study, based on 3817 women in China, reported a 3.4-fold increase in the risk of delivering a LBW infant with serum zinc <560 µg/L (adjusted RR: 3.41, 95% CI: 1.97, 5.91) [56]. This is in contrary to two studies that reported significantly higher zinc concentrations in women with an SGA infant in the third trimester [47,55]. However, these findings were based on a relatively small number of women: 40–51 pregnant women including 10–16 women with SGA. Conversely, another study, which followed 476 women of whom 39 gave birth to an SGA infant, found the incidence of LBW to be 8 times higher in women with serum zinc in the lowest quartile (457.5–797.4 µg/L) compared to the highest (1039.2–1660.1 µg/L) (8.2, 95% CI: 2.4–27.5) [52]. The remaining eight studies found no differences in maternal zinc concentrations between women with a SGA infant and those with an uncomplicated pregnancy. However, one study found a positive correlation between maternal zinc status and birthweight (*r* = 0.632, *p* ˂ 0.001) [50].

The association between maternal circulating zinc and birthweight was assessed in 14 studies based on women where inadequate dietary zinc intake was predicted to affect ≥17% of the population [57,58,59,60,61,62,63,64,65,66,67,68,69], of which 7 reported a significant association (Table 1) [57,59,60,61,66,67,68]. All three of the studies based on women from Africa reported serum/plasma zinc on average 72–333 µg/L lower in women who gave birth to a LBW infant compared to those who gave birth to an appropriate weight infant [57,59,67]. In another study, the risk of delivering a LBW infant was also reported to be 3-fold greater in women with serum zinc levels ≤392.2 µg/L compared to those with levels above this figure (3.07, 95% CI: 1.07–8.97) [67]. Conversely, two other studies reported serum zinc to be 40–172 µg/L higher in women who gave birth to a LBW infant compared to those who gave birth to an appropriate weight infant [60,66]. A further four studies, also based on women from India, reported no association between circulating zinc levels and birthweight [62,63,64,69] and this was also reported in two studies of Turkish women [58,65]. However, univariate analysis and small sample size in these studies may not provide an accurate assessment of the effects of maternal circulating zinc and birthweight.

### 3.2. Hypertensive Disorders of Pregnancy

Only one study assessed dietary zinc intake and the association with hypertensive disorders (Table 2) and found no significant differences in dietary zinc intake between 13 women who developed a hypertensive disorder in pregnancy and 44 whose pregnancies remained uncomplicated [70].

Thirteen studies analyzed serum/plasma zinc in women who developed a hypertensive disorder of pregnancy in women residing in countries where inadequate zinc intake is estimated to be low (<17%) (Table 2). Three studies reported mean serum/plasma zinc to be on average 120–1200 µg/L lower in women who developed PE compared to women whose pregnancies remained uncomplicated [49,71,72] and included one study that reported a reduction in risk of PE with serum levels above 1360 µg/L after adjusting for maternal age, height and weight before pregnancy (aOR: 0.005, 95% CI: 0.001–0.07) [71]. A further two studies reported circulating zinc to be lower in women who developed severe PE (blood pressure BP ≥ 160/110) compared to women whose pregnancies remained uncomplicated [73,74]. The remaining eight studies, whose sample sizes ranged from 10–271 women with PE/GH and 10–2038 women with an uncomplicated pregnancy, reported no difference in maternal zinc status between women with a hypertensive disorder of pregnancy and those without [38,47,54,75,76,77,78,79].

There were twenty studies that analyzed circulating zinc in women with a hypertensive disorder of pregnancy in populations where inadequate zinc intake is estimated to be ≥17% (Table 2) [32,33,34,36,37,39,80,81,82,83,84,85,86,87,88,89,90,91,92,93]. Ten studies reported mean serum/plasma zinc to be significantly lower in women who developed PE and/or GH [33,34,36,37,39,80,81,82,83,93] however, one reported plasma zinc to be higher in women with PE compared to those whose pregnancies remained uncomplicated when measured during the latent phase of labor; with (PE mean (SD): 15.53 (4.92) vs. uncomplicated: 11.93 (3.11) μg/g protein, *p* = 0.003) [89]. These studies also included three which found circulating zinc to be 80–260 μg/L lower in women who developed severe PE when compared to women whose pregnancies remained uncomplicated [37,39,83]. A further nine studies reported no difference in circulating zinc between women with PE/GH and those whose pregnancies remained uncomplicated.

### 3.3. Spontaneous Preterm Birth

The literature search identified four studies which measured dietary zinc intakes during pregnancy and sPTB with varying conclusions (Table 3) [43,44,94,95]. Two of these studies, which analyzed 5738 and 818 women respectively, determined that low zinc intake (≤6 mg/day which is ≤54% of the recommended 11 mg/day [21]) was associated with a more than 2-fold increase in the risk of delivering preterm (aOR: 2.3, 95% CI: 1.2–4.5 and aOR: 1.85 95% CI: 1.09–3.12, respectively), after adjusting for factors such as ethnicity, pre-pregnancy BMI, smoking, alcohol and multivitamin consumption [44,94]. If delivery date was calculated by last menstrual period (LMP), zinc intake below 9 mg/day was associated with a 2.75-fold increased risk in delivering <32 weeks gestation (aOR: 2.75, 95% CI: 1.31–5.77) [44]. However, another study reported no association between low dietary zinc intake (less than the median) and the risk of sPTB (OR: 1.1, 95% CI: 0.7–1.7) [43] but mean zinc intake of the women in this study was 14 mg/day, higher than the recommended 11 mg/day, indicating that low zinc intake was not prevalent within this studied population.

When separated based on estimates of inadequate zinc intake, there were three studies which assessed whether there was an association between circulating zinc and sPTB in low-risk populations (Table 3). While two showed no significant difference between serum/plasma zinc levels during gestation in women who gave birth preterm and those who gave birth at term [48,54], one study which recruited 3081 women in China found a 2.4-fold increase risk of PTB with serum levels <767 µg/L (aOR: 2.41, 95% CI: 1.57, 3.70) [96].

The association between maternal circulating zinc and sPTB was determined in four studies on populations with inadequate zinc intake ≥17%, all of which sampled women in India (Table 4) [61,63,64,69]. Two of the studies reported serum/plasma zinc to be higher in women who delivered preterm compared to those who delivered at term (average 98–1991 µg/L increase) [63,64]. However, no difference in circulating zinc measured at delivery was reported in the remaining two studies [61,69].

### 3.4. Gestational Diabetes Mellitus

Two studies looked at the association between dietary zinc intake and GDM (Table 4) [97,98]. One collected data at 24–28 weeks gestation, and found an 11% reduction in the risk of gestational hyperglycaemia with every 1 mg/day increase in dietary zinc intake (aOR: 0.89, 95% CI: 0.82–0.96) [97]. The second, which sampled women at 14–20 weeks’ gestation, found no association between maternal dietary zinc intakes below 50% of the recommended daily allowance and GDM (OR: 1.4, 95% CI: 0.6–2.9) [98]. Differences between the studies included when dietary zinc was measured (early versus late second trimester) as well as ethnicity (Italian versus Iranian in which, genetic and cultural differences are likely).

Of the five studies which assessed the association between circulating zinc and GDM in countries where inadequate zinc intake is estimated to be <17%, two, both studying Italian women, reported a significant difference in serum/plasma zinc in women who developed GDM compared to women whose pregnancies remained uncomplicated (Table 4) [47,97]. However, while one study reported that serum zinc was negatively associated with the risk of hyperglycemia in pregnancy (aOR: 0.94, 95% CI: 0.91–0.96) [97], the other found that there was in increase in serum zinc in women with GDM compared to women whose pregnancy remained uncomplicated (GDM mean (SD): 766.6 (117.6) vs. uncomplicated: 627.5 (150) µg/L, *p* ˂ 0.001) [47]. Both studies sampled women at similar times during pregnancy and used atomic absorption spectrometry to quantitate zinc. The remaining three studies found no difference in circulating zinc [35,38,99] however, given the small sample size of women with GDM in these studies (*n* = 5–46), it is likely they were underpowered and not suitable for the chosen statistical tests.

There were two studies that sampled women from countries where inadequate zinc intake was estimated to be ≥17% and assessed the association between maternal circulating zinc and GDM (Table 4) [98,100]. Neither study reported a difference in serum zinc in early pregnancy or at delivery in women with GDM compared to those whose pregnancies remained uncomplicated.

## 4. Discussion

This systematic review assessed whether maternal circulating zinc levels and/or dietary zinc intake were associated with a number of pregnancy complications. Overall, the evidence regarding the association between maternal zinc status and PE/GH, LBW/SGA, sPTB and GDM is weak and heterogeneity between the studies made comparisons difficult. However, systematic analysis of the available literature indicated some trends between maternal zinc status and infant birthweight as well as the development of severe PE (BP ≥160/110 mmHg). 

There is consistent evidence in animal models that maternal dietary zinc deficiency during pregnancy reduces fetal growth [16,17,18,19]. From the studies that measured maternal zinc intake during pregnancy reviewed here, a possible relationship between low zinc intake (≤54% of the recommended 11 mg/day) and decreased infant birthweight may exist in human populations. Both food frequency questionnaires and 24 h recalls are limited by the preparedness of the participants to accurately record their diets, the food composition tables used and their ability to capture variations within diets [102]. This may explain the conflicting results between studies which assessed dietary zinc intake and the association with infant birthweight, sPTB and GDM. However, three of the four studies that measured dietary zinc intake in pregnancy and recorded infant birthweight reported a significant reduction in maternal zinc status in those who delivered a LBW/SGA infant [42,44,45]. The relationship between infant birthweight and maternal serum/plasma zinc is less clear. Plasma measures of zinc are considered preferable over serum as erythrocytes can be a source of zinc contamination within serum samples [22]. However, plasma zinc only accounts for approximately 0.1% of total body zinc [103], is heavily influenced by confounding factors like stress, infection and hormones [101,104,105,106,107] and does not directly correlate with dietary zinc intake [108]. This limits how useful measuring circulating zinc is as a biomarker for health and disease. When studies on LBW/SGA that measured maternal circulating zinc were separated based on populations where inadequate zinc intake is predicted to be ≥17%, 7 of the 13 studies reported a difference in serum/plasma zinc between women who delivered LBW/SGA infant and those whose infants were of an appropriate weight. Given the lack of suitable alternatives, particularly in studies of pregnant women, determining zinc status by measuring serum/plasma zinc can still be informative about the importance of zinc to pregnancy, especially if measured in conjunction with dietary zinc intakes.

Other maternal factors such as age, BMI, smoking status and alcohol consumption in pregnancy not only influence pregnancy outcome but also circulating zinc [109,110]. BMI is a significant factor in influencing the risk for developing PE and GH [111,112]. However, only 11 of the 32 studies on PE/GH [33,36,54,71,75,79,80,82,84,88,92] reported on BMI, making it difficult to comment on whether differences in BMI may be influencing the outcomes of the studies included in this review. Despite this, there may be a relationship between maternal circulating zinc levels and the severity of PE. Mean maternal zinc concentrations in women with severe PE (ranging from 388 to 410 μg/L) [73,74,83] were well below 562.1 μg/L, which is the defined zinc deficiency cut-off [26,113]. In women with mild PE and those with uncomplicated pregnancies, mean maternal zinc concentrations ranged between 684–831 µg/L [37,39,83] and 630–1022 µg/L [37,39,74,83] respectively. A current leading hypothesis relating to the development of PE is increased placental oxidative stress [114]. Zinc itself has antioxidant capabilities and is an integral structural component of superoxide dismutase, a first line defense antioxidant [115] which has reduced activity in cell lines, animal models and human studies of zinc deficiency [116,117,118,119,120]. Hence, it is possible in pregnancies complicated by PE, that low maternal zinc concentration (˂562.1 µg/L) may reduce the potential to combat rises in free radical production and increase the severity of the complication.

Zinc levels in maternal circulation decrease across gestation; this is thought to be due to a combination of increased maternal blood volume and fetal demands [40,121,122,123], and therefore comparisons between studies which measured zinc in maternal serum or plasma early in pregnancy versus late should be interpreted with caution. Overall, regardless of pregnancy outcome, the majority (31 out of 59 studies which measured maternal circulating zinc) collected samples during labor or at delivery. Physiologically, parturition results in huge changes to maternal hormonal profile with rises in estrogen, oxytocin and prostaglandin required to initiate labor [124]. Furthermore, there is an increase in the production of inflammatory cytokines and a withdrawal of anti-inflammatory cytokines within the gestational tissues [125]. Infection and inflammation decrease plasma zinc [104] and use of the contraceptive pill, which raises estrogen and progesterone levels, also decreases circulating zinc [101,105]. Given that pregnancy itself is likely to confound zinc status, this has implications for interpreting studies that have measured serum/plasma zinc at delivery. In addition, how zinc may be associated with a pregnancy outcome needs to be measured before the pregnancy complication has manifested. Only five studies of 6795 pregnant women in total measured either circulating zinc or dietary zinc intake prior to 20 weeks gestation [44,53,54,79,98]. All found no significant difference in maternal zinc status during this time period between women who developed a pregnancy complication and those who did not, indicating that zinc status in early pregnancy may not be associated with adverse pregnancy outcomes.

Due to the additional demands associated with pregnancy and fetal growth, pregnant women are more vulnerable to multiple nutrient deficiencies [126] and this is potentially another cofounding factor when assessing the association between maternal zinc status and pregnancy outcome. This is because nutrients can interact with each other in both a positive (e.g., vitamin A and zinc [127]) and negative manner (e.g., calcium or iron and zinc [128,129]). A number of studies reviewed here measured serum/plasma concentrations of other nutrients as well as zinc, including copper [35,97], iron [75,98], selenium [51,88], magnesium [95,99] and lead [78]. While circulating zinc levels were not different for the pregnancy outcomes studied in these articles, those of other micronutrients were. Serum copper concentrations were found to be higher in women with GDM or those who delivered an SGA infant when compared to women with an uncomplicated pregnancy in two studies [35,97]. Furthermore, serum iron was higher in women with PE and GDM compared to women whose pregnancies were uncomplicated [75,98]. Two other studies found selenium to be lower in the serum of women with PE or those who delivered an SGA infant compared to women with an uncomplicated pregnancy [51,88]. Therefore, it is important to consider other nutritional factors that may influence pregnancy outcome as well as micronutrient ratios in order to fully understand the importance of micronutrient status on pregnancy success.

Finally, the lack of studies identified in this review analyzing truly zinc deficient women, nor those in populations at high risk of zinc deficiency, is a major limitation in determining the effects of zinc on pregnancy outcome. Only 8 of the 64 studies reported mean circulating zinc below 562.1 μg/L [49,50,60,72,74,83,87,91] and there were very few studies based on women in countries where inadequate zinc intake is predicted to be prevalent like South-East Asia and parts of Africa [26,27]. The majority of studies were based on populations in the United States and Europe where zinc deficiency is estimated to only affect 3.9%–12.7% of the population [26]. Therefore, there is the potential that the results from this review may be skewed given the lack of evidence based on women living in areas predicted to be at high risk of zinc deficiency.

## 5. Conclusions

The current review has explored the connection between maternal zinc status and pregnancy complications including hypertensive disorders of pregnancy, infant birthweight, spontaneous preterm birth (sPTB) and gestational diabetes mellitus (GDM). While it appears that there may be a relationship between maternal dietary zinc intake and infant birthweight and the development of severe PE, there is little evidence to suggest an association between zinc and sPTB or GDM. However, heterogeneity in the studies identified in this review reflects real uncertainty in the evidence linking zinc deficiency and pregnancy complications and therefore this warrants further study, particularly in developing countries whose populations are at increased risk of zinc deficiency. If we are to continue to reduce preventable deaths of newborns and children under the age of five [6], understanding the importance of micronutrients like zinc in child development, particularly in utero, will greatly increase the likelihood of success. Future studies need to focus on women more vulnerable to zinc deficiency in pregnancy in order to fully determine the effects of zinc status on pregnancy outcome. 

## Figures and Tables

**Table 1:** Included studies assessing maternal zinc status and birthweight.

Author, Country	Sample Size	Zinc Measure	Outcome of the Study
		(1) Sample Type	
		(2) Time at Which Gestation Diet Was Assessed or Sample Collected	
		(3) Method of Analysis	
[42] Simmer, United Kingdom ^a^	28 SGA 29 uncomplicated	Dietary zinc intake Third trimester of pregnancy 7 day dietary recall	↓ mean (SEM) dietary intake in the SGA mothers compared to the women with uncomplicated pregnancies. **SGA: 11.3 (0.5) vs. uncomplicated: 13.0 (0.6) mg/day, *p* < 0.05**
[43] Negandhi, India ^b^	144 LBW 240 uncomplicated	Dietary zinc intake 26–30 weeks 24 h dietary recall	↓ mean dietary zinc intake in women with a LBW infant compared to those with an uncomplicated pregnancy. **LBW: 5.39 mg/day vs. uncomplicated 6.77 mg/day, *p* < 0.001**
[44] Scholl, United States ^c^	115 with zinc intake ≤6 mg/day 699 with zinc intake ˃6 mg/day	Dietary zinc intake 28 and 36 weeks 24 h dietary recall	2-fold ↓ risk of delivering a LBW infant with dietary zinc intake ˃6 mg/day. **OR: 2.01, 95% CI: 1.11–3.66**
[45] Neggers, United States ^d^	180 LBW 1218 uncomplicated	Dietary zinc intake 18 and 30 weeks 24 h dietary recall using the nutrient data base developed by the University of Minnesota	NS association between low dietary zinc intake (less than median) and risk of LBW. OR: 1.4, 95% CI: 0.9–2.1
*Inadequate dietary zinc intake estimated to affect <17% of the studied population*			
[46] Wang, China ^b^	247 with serum zinc <560 µg/L 2940 with serum zinc ≥560 µg/L	Fasting serum zinc Across gestation Flame AAS	↑ incidence of LBW in the mothers with serum zinc <560 µg/L compared to those with serum zinc ≥560 µg/L. **Adjusted RR: 3.41, 95% CI: 1.97, 5.91**
[47] Voss Jepsen, Denmark ^a^	10 SGA 30 uncomplicated	Heparin plasma zinc Collected at 35–41 weeks AAS	↑ mean (SD) plasma zinc between SGA mothers and those with uncomplicated pregnancies. **SGA: 732 (85) vs. uncomplicated: 654 (78) μg/L, *p* = 0.03**
[48] Borella, Italy ^a^	16 SGA 35 uncomplicated	Heparin plasma zinc Collected in the third trimester Flame AAS	↑ mean (SD) plasma zinc in SGA women compared to women with uncomplicated pregnancies. **SGA: 685.6 (119.6) vs. uncomplicated: 627.5 (150) µg/L, *p* < 0.001**
[49] Neggers, USA ^e^	39 LBW 437 uncomplicated	Serum zinc Collected across gestation Flame AAS	8-fold ↑ prevalence of LBW with serum zinc in the lowest quartile (457.5–797.4 µg/L) compared to the highest (1039.2–1660.1 µg/L). **OR: 8.2, 95% CI:2.4–27.5**
[50] Bro, Denmark ^a^	47 SGA and 34 preterm 220 uncomplicated	Serum zinc Collected at delivery Flame AAS	NS mean (SD) serum zinc levels between SGA and women with uncomplicated pregnancies. SGA: 764.7 (119.6) vs. uncomplicated: 679.7 (98) µg/L
[38] Hyvonen-Dabek, Finland ^f^	4 SGA 10 uncomplicated	Serum zinc Collection time not specified Particle induced X-ray emission	NS mean (SD) serum zinc in SGA women compared to those with uncomplicated pregnancies. SGA: 1270 (320) vs. uncomplicated: 1150 (220) µg/L
[51] Mistry, UK ^a^*	19 SGA 107 uncomplicated	Heparin plasma zinc Collected at 28–32 weeks Inductively coupled plasma mass spectrometry	NS in mean (95% CI) plasma zinc between SGA women and those with uncomplicated pregnancies. SGA: 708.1 (510.4–905.8) vs. uncomplicated: 634.4 (580.5–688.2) μg/L
[52] Tamura, USA ^g^	80 SGA 80 uncomplicated	Serum zinc Collected at 18 weeks and 30 weeks Flame AAS	NS in mean (SD) plasma zinc between SGA and women with uncomplicated pregnancies at 18 weeks. SGA: 627 (118) vs. uncomplicated: 667 (98) µg/L NS in mean (SD) plasma zinc between SGA and women with uncomplicated pregnancies at 30 weeks. SGA: 562 (92) vs. uncomplicated: 575 (92) µg/L
[53] Tamura, USA ^a^	139 SGA 2038 uncomplicated	Non-fasting heparin plasma zinc Collected at first prenatal visit (6 to 34 weeks) Flame AAS	NS in the prevalence (*n* (%)) of SGA measured between the lowest quartile and upper 3 quartiles of zinc. Highest: 103 (4.4) vs. lowest: 36 (4.8)
[54] Ghosh, China ^a^	22 SGA 38 uncomplicated	Serum zinc Collected within 24 h of delivery AAS	NS in mean (SD) serum zinc levels between SGA and women with uncomplicated pregnancies. SGA: 508.1 (185.9) vs. uncomplicated: 542.3 (162.8) μg/L
[55] Cherry, USA ^b^	29 LBW 230 uncomplicated	Heparin plasma zinc Collected across gestation AAS	NS mean (SEM) plasma zinc in mothers with a LBW infant compared to mothers with uncomplicated pregnancies. LBW: 604.9 (22.4) vs. uncomplicated: 577.2 (7.7) μg/L
[56] Bogden, USA ^h^	22 LBW 50 uncomplicated	EDTA plasma zinc Collected at delivery Flame AAS	NS mean (SEM) plasma zinc in women with a LBW infant compared to women with uncomplicated pregnancies. LBW: 640 (20) vs. uncomplicated: 620 (20) µg/L
*Inadequate dietary zinc intake estimated to affect ≥17% of the studied population*			
[57] Atinmo, Nigeria ^h^	20 LBW 30 uncomplicated	Heparin plasma zinc Collected at delivery AAS	↓ mean (SD) serum zinc in women with a LBW infant compared to those with uncomplicated pregnancies. **LBW: 663.1 (144.6) vs. uncomplicated: 731.5 (235.6) µg/L, *p* < 0.05**
[58] Abass, Sudan ^b^	50 LBW 50 uncomplicated	Serum zinc AAS Atomic absorption spectrometry	↓ median (IQR) serum zinc in women with a LBW infant compared to those with uncomplicated pregnancies. **LBW: 629 (363–968) vs. uncomplicated 962 (846–1257) µg/L, *p* < 0.001**
[59] Rwebembera, Tanzania ^c^	81 LBW 84 uncomplicated	EDTA plasma zinc Collected at delivery Flame AAS	3-fold ↓ risk of delivering a LBW infant with serum zinc ≥ 392.2 µg/L **OR: 3.07, 95% CI: 1.07–8.97**
[60] Bahl, India ^c^	19 LBW 56 uncomplicated	Serum zinc Collected at delivery Flame AAS	↓ mean (SD) serum zinc in women with a LBW infant compared to those with uncomplicated pregnancies. **LBW: 553 (43) vs. 692 (95) µg/L, *p* < 0.001**
[61] Singh, India ^e^	47 LBW 45 uncomplicated	Serum zinc Collected at delivery AAS	↓ mean (SD) serum zinc in women with a LBW infant compared to those with uncomplicated pregnancies. **LBW: 623 (330) vs. uncomplicated: 895 (514) μg/L, *p* < 0.001**
[62] Prema, India ^e^	23 LBW 208 uncomplicated	Serum zinc Collected at delivery between 9–11.30 a.m. Flame AAS	↑ mean (SD) serum zinc in mothers with a LBW infant compared to mothers with an uncomplicated pregnancy. **LBW: 660 (162) vs. uncomplicated: 620 (146) µg/L, *p* < 0.01**
[63] Badakhsh, Iran ^b^	30 LBW 110 uncomplicated	Serum zinc Collected at delivery AAS	↑ mean (SD) serum zinc in mothers with a LBW infant compared to mothers with an uncomplicated pregnancy. **LBW: 686.2 (204.8) vs. uncomplicated: 514.3 (138.8) µg/L, *p* < 0.001**
[64] Goel, India ^a^	20 LBW 25 uncomplicated	Heparin plasma zinc Collected at delivery AAS	NS mean (SD) plasma zinc in women with a LBW infant compared to those with an uncomplicated pregnancy. LBW: 726 (61) vs. uncomplicated: 763 (56) μg/L
[65] Srivastava, India ^b^	26 LBW 25 uncomplicated	Heparin plasma zinc Collected at delivery Flame AAS	NS mean (SD) plasma zinc between mothers with a LBW infant and mothers with uncomplicated pregnancies. LBW: 6470 (4860) vs. uncomplicated: 5670 (2490) µg/L
[66] Jeswani, India ^a^	10 SGA 25 uncomplicated	Serum zinc Collected at 28–40 weeks AAS	NS mean (SD) serum zinc in SGA women compared to those with uncomplicated pregnancies. SGA: 938 (76.2) vs. uncomplicated: 962.8 (194.8) µg/L
[67] George, India ^a^	65 SGA 51 uncomplicated	Heparin plasma zinc Collected before labor between 8–10 a.m. AAS	NS in mean (SD) plasma zinc between SGA and women with uncomplicated pregnancies. SGA: 675 (90) vs. uncomplicated: 706.7 (139) µg/L
[68] Akman, Turkey ^f^	22 SGA 34 uncomplicated	Serum zinc Collected at delivery AAS	NS mean (SD) serum zinc between SGA women and women with uncomplicated pregnancies. SGA: 1218 (543) vs. uncomplicated 1038 (343) µg/L
[69] Ozdemir, Turkey ^b^	16 LBW 59 uncomplicated	Serum zinc Collected at 38–42 weeks Flame AAS	NS mean (SD) serum zinc between mothers with a LBW infant and mothers with uncomplicated pregnancies. Data represented on graphs

^a^ SGA defined as ˂10th percentile; ^b^ LBW defined as ˂2500 g; ^c^ LBW defined as ≤2000 g; ^d^ LBW defined as ˂2750 g; ^e^ LBW defined as ˂2000; ^f^ SGA not defined; ^a^* SGA defined as ˂10th percentile based on customised centiles; ^g^ SGA defined as ˂15th percentile; ^h^ LBW defined as ≤2500 g. **Bold print signifies results that were significantly different**. Abbreviations: AAS: atomic absorption spectrometry; CI: confidence interval; IQR: interquartile range; LBW: low birth weight; NS: non-significant; OR: odds ratio; SD: standard deviation; SEM: standard error of the mean; SGA: small for gestational age.

**Table 2:** Included studies assessing maternal zinc status and hypertensive disorders of pregnancy.

Author, Country	SAMPLE SIZE	Zinc Measure	Outcome of the Study
		(1) Sample Type	
		(2) Time at Which Gestation Diet Was Assessed or Sample Collected	
		(3) Method of Analysis	
[70] Tande, United States ^a,b^	13 hypertensive (11 PE + 2 GH) 44 uncomplicated	Dietary and supplement intake First 3 months of pregnancy Harvard food frequency questionnaire	NS in mean (SEM) dietary zinc intake between those with and without gestational hypertension. Hypertensive: 16.9 (1.56) vs. uncomplicated: 15.4 (1.03) mg/day
*Inadequate dietary zinc intake estimated to affect <17% of the studied population*			
[71] Lazebnik, United States ^a,b^	17 PE and 14 hypertensive 31 uncomplicated	Plasma zinc Collected within 1 h of delivery AAS	↓ mean (SD) serum zinc in women with PE when compared to women with uncomplicated pregnancies. **PE: 420 (100) vs. uncomplicated: 520 (130) µg/L, *p* < 0.05** NS mean (SD) plasma zinc in hypertensive women compared to those whose pregnancies remained uncomplicated. Hypertensive: 530 (110) vs. uncomplicated: 520 (110) µg/L
[55] Cherry, United States ^a^	48 toxemic/ hypertensive 207 uncomplicated	Heparin plasma zinc Collected across gestation AAS	↓ mean (SEM) plasma zinc in women with toxemia/ hypertension compared to women with uncomplicated pregnancies. **Toxemic: 541.5 (16.8) vs. uncomplicated: 590.7 (8) μg/L, *p* < 0.009**
[72] Kim, Korea ^a^	29 PE 30 uncomplicated	Serum zinc Collected at delivery Instrumental neutron activation analysis	↓ mean (SEM) serum zinc in mothers with PE compared to women with uncomplicated pregnancies. **PE: 700 (200) vs. uncomplicated: 1900 (500) μg/L, *p* < 0.0001**
[73] Kiilholma, Finland ^c,d^	10 mild PE and 10 severe PE 20 uncomplicated	Serum zinc Collected at delivery Particle induced X-ray emission	↓ mean (SD) serum zinc in women with mild and severe PE compared to women with uncomplicated pregnancies. **Mild PE: 510 (70) and severe PE: 370 (10) vs. uncomplicated: 630 (90) μg/L, *p* < 0.001 for both, respectively** ↓ mean (SD) serum zinc in women with severe PE compared to those with mild PE. **Severe PE: 370 (10) vs. mild PE: 510 (70) μg/L, *p* < 0.005**
[74] Araujo Brito, Brazil ^e^	20 mild PE and 24 severe PE 50 uncomplicated	Fasting sodium citrate plasma zinc Collected before delivery Flame AAS	↓ mean (SD) plasma zinc in mothers with severe PE compared to mothers with uncomplicated pregnancies. **Severe PE: 388 (82) vs. uncomplicated: (483 (83) µg/L, *p* < 0.05** NS mean (SD) plasma zinc in women with mild PE compared to women with uncomplicated pregnancies. Mild PE: 500 (94) vs. uncomplicated: (483 (83) µg/L
[75] Magri, Malta ^b^	33 GH 110 uncomplicated	Serum zinc Collected in third trimester Electro-thermal AAS	NS in mean (SD) serum zinc between women with GH and women with uncomplicated pregnancies. PE: 606 (80) vs. uncomplicated: 636 (100) μg/L
[76] Fenzl, Croatia ^a,b^	30 PE and 30 GH 37 uncomplicated	Fasting serum zinc Collected at the time of diagnosis Flame AAS	NS in mean (SD) serum zinc between both women with PE or GH women and women with uncomplicated pregnancies. PE: 603 (93) and GH: 599 (83) vs. uncomplicated: 578 (93) μg/L
[77] Katz, Israel ^d^	43 severe PE 80 uncomplicated	Plasma zinc Collected immediately after delivery Inductively coupled plasma mass spectrometry	NS mean (SD) plasma zinc in mothers with severe PE vs. mothers with uncomplicated pregnancies. Severe PE: 685 (875) vs. uncomplicated: 534 (139) µg/L
[38] Hyvonen-Dabek, Finland ^f^	10 hypertensive 10 uncomplicated	Serum zinc Collection time not specified Particle induced X-ray emission	NS mean (SD) serum zinc in women with PE compared to women with an uncomplicated pregnancy. PE: 1070 (320) and hypertensive: 1090 (170) vs. uncomplicated: 1150 (220)
[48] Borella, Italy ^a^	24 hypertensive 35 uncomplicated	Heparin plasma zinc Collected in the third trimester Flame AAS	NS mean (SD) plasma zinc in the hypertensive women compared to those who remained uncomplicated. Hypertensive: 685.6 (149) vs. uncomplicated: 627.5 (150) µg/L
[78] Mistry, United Kingdom ^a^	244 PE 472 uncomplicated	Non-fasting heparin plasma zinc Collected at 15 weeks gestation Inductively coupled plasma mass spectrometry	NS median (interquartile range) plasma zinc in women with PE women compared to those with uncomplicated pregnancies. PE: 579.6 (521.1–638.6) vs. uncomplicated: 575.7 (515.6–641.7) µg/L
[53] Tamura, United States ^a^	271 hypertensive 2038 uncomplicated	Non-fasting heparin plasma zinc Collected at first prenatal visit (6 to 34 weeks) Flame AAS	NS in the prevalence (*n* (%)) of hypertension measured between the lowest quartile and upper 3 quartiles of zinc. Highest: 205 (7.9) vs. Lowest: 66 (7.7)
[79] Lao TT, China ^a^	28 PE 28 uncomplicated	Heparin plasma zinc Collected after diagnosis, before delivery Flame AAS	NS mean (SD) plasma zinc in women with PE compared to women with uncomplicated pregnancies. PE: 641 (163) vs. uncomplicated: 647 (111) µg/L
*Inadequate dietary zinc intake estimated to affect ≥17% of the studied population*			
[80] Sarwar, Bangladesh ^a^	50 PE 58 uncomplicated	Fasting serum zinc Collected ˃20 weeks gestation Flame AAS	↓ mean (SEM) serum zinc in mothers with PE compared to mothers with uncomplicated pregnancies. **PE: 770 (50) vs. uncomplicated: 980 (30) µg/L, *p* < 0.001**
[34] Kumru, Turkey ^a^	30 PE 30 uncomplicated	Serum zinc Collection time not specified AAS	↓ mean serum zinc in women with PE when compared to women with uncomplicated pregnancies. **Data represented on graphs, *p* < 0.001**
[81] IIhan, Turkey ^a^	21 PE 20 uncomplicated	Serum zinc Collected at 31–38 weeks Flame AAS	↓ mean (SD) serum zinc in women with PE when compared to those with an uncomplicated pregnancy. **PE: 829.4 (289.3) vs. uncomplicated: 1251.9 (242.3) μg/L, *p* < 0.001**
[82] Bakacak, Turkey ^a^	38 PE 40 uncomplicated	Fasting serum zinc 32–38 weeks Flame AAS	↓ median (max-min) serum zinc in women with PE when compared to those with an uncomplicated pregnancy. **PE: 812.4 (1106.5–624) vs. uncomplicated: 1084.5 (1385.5–881.2) μg/L, *p* < 0.001**
[36] Farzin, Iran ^a^	60 PE 60 uncomplicated	Fasting heparin plasma zinc Collection time not specified Flame AAS	↓ mean (SEM) serum zinc in mothers with PE compared to mothers with uncomplicated pregnancies. **PE: 764.9 (176.2) vs. uncomplicated: 1006.1 (201.2) µg/L, *p* < 0.001**
[83] Al-Jameil, Saudi Arabia ^a^	40 PE 40 uncomplicated	Serum zinc Collected in the third trimester Inductively coupled plasma optical emission spectrometry	↓ mean (SD) serum zinc in mothers with PE compared to mothers with uncomplicated pregnancies. **PE: 670 (590) vs. uncomplicated: 1300 (830) µg/L, *p* < 0.05**
[33] Akinloye, Nigeria ^a^	49 PE 40 uncomplicated	Serum zinc Collection time not specified Flame AAS	↓ mean (SD) serum zinc between women with PE and women with uncomplicated pregnancies. **PE: 562 (92) vs. uncomplicated: 614 (52) μg/L, *p* < 0.05**
[39] Jain, India ^e^	25 mild PE and 25 severe PE 50 uncomplicated	Serum zinc Collection time not specified AAS	↓ mean (SD) serum zinc between women with mild PE and those with uncomplicated pregnancies. **Mild PE: 831 (111) vs. uncomplicated: 1022 (157) μg/L, *p* < 0.05** ↓ mean (SD) serum zinc between women with severe PE and women with uncomplicated pregnancies. **Severe PE: 787 (92) vs. uncomplicated: 1022 (157) μg/L, *p* < 0.05**
[37] Gupta, India ^b,e^	47 mild PE and. 18 severe PE and 10 eclamptic 74 uncomplicated	Non-fasting heparin plasma zinc Collection time not specified AAS	↓ mean (SD) serum zinc in mothers with severe PE and eclampsia compared to mothers with uncomplicated pregnancies. **Severe PE: 607 (107) and eclampsia: 607 (171) vs. uncomplicated: 695 (119) μg/L, *p* < 0.01** NS in mean (SD) serum zinc between women with mild PE and women with uncomplicated pregnancies. Mild PE: 684 (134) vs. uncomplicated: 695 (119) μg/L
[84] Bassiouni, Egypt ^g,d^	52 PE (28 mild and 24 severe) 20 uncomplicated	Heparin plasma zinc Collected at delivery AAS	NS in mean (SD) plasma zinc in women with mild PE compared to women with uncomplicated pregnancies. **Mild PE: 604.2 (162.7) vs. uncomplicated: 646 (173.7) μg/L** ↓ mean (SD) plasma zinc in women with severe PE compared to the women with uncomplicated pregnancies. **Severe PE: 410.8 (116.5) vs. uncomplicated: 646.0 (173.7 μg/L, *p* < 0.001**
[85] Harma, Turkey ^a^	24 PE 44 uncomplicated	Heparin plasma zinc Collected just during the latent phase of labor AAS	↑ mean (SD) plasma zinc levels in women with PE when compared to women with uncomplicated pregnancies. **PE: 15.53 (4.92) vs. uncomplicated: 11.93 (3.11) μg/g protein, *p* = 0.003**
[86] Rafeeinia, Iran ^h^	35 PE and 15 severe PE 50 uncomplicated	Fasting serum zinc Collected in the third trimester AAS	NS mean (SD) serum zinc in mothers with PE or severe PE and uncomplicated pregnancies. Mild PE: 690 (40) and severe PE: 780 (80) vs. uncomplicated: 720 (40) µg/L
[87] Vafaei, Iran ^e^	20 mild PE and 20 severe PE 40 uncomplicated	Serum zinc Collected at 28–40 weeks Auto-analyser	NS mean (SD) serum zinc in either the mild or severe PE women compared to women with uncomplicated pregnancies. Data represented on graphs
[88] Ahsan, Bangladesh ^a,i^	44 PE and 33 eclampsia 27 uncomplicated	Serum zinc Collected at 28–42 weeks Flame AAS	NS mean (SD) serum zinc in PE or eclamptic women compared to women with uncomplicated pregnancies. PE: 1045.8 (131) and eclampsia: 915 (131) vs. uncomplicated: 980.4 (131) µg/L
[89] Rathore, India ^a^	14 PE 47 uncomplicated	Serum zinc Collected at delivery Flame AAS	NS mean (SD) serum zinc between women with PE and those with uncomplicated pregnancies. PE: 492 (178) vs. uncomplicated: 575 (216) μg/L
[90] Kolusari, Turkey ^a^	47 PE 48 uncomplicated	Serum zinc Collected between 29 and 38 weeks AAS	NS mean (SD) serum zinc between women with PE women and those with uncomplicated pregnancies. PE: 10.6 (4.4) vs. uncomplicated: 12.7 (4.1) µg/L
[91] Atamer, Turkey ^a^	32 PE 28 uncomplicated	Fasting serum zinc Collected at 28–29 weeks Flame AAS	NS in mean (SD) serum zinc between women with PE and women with uncomplicated pregnancies. PE: 792 (180) vs. uncomplicated: 1086 (199) μg/L
[92] Adam, Turkey ^a^	20 PE 20 uncomplicated	Plasma zinc Collected before the onset of labor Flame AAS	NS mean (SD) plasma zinc in women with PE compared to women with an uncomplicated pregnancy. PE: 313 (47) vs. uncomplicated: 341 (44) µg/L
[93] Vigeh, Iran ^a^	31 PE 365 uncomplicated	Heparin plasma zinc Collected at delivery Inductively coupled plasma mass spectrometry	NS mean (SD) plasma zinc between women with PE women and women with uncomplicated pregnancies. PE: 5200 (1444) vs. uncomplicated: 5561 (1057) µg/L
[32] Adeniyi, Nigeria ^a^	55 pregnant women	Plasma zinc Collection time not specified AAS	NS mean (SD) plasma zinc in women with PE compared to women with uncomplicated pregnancies. PE: 940 (270) vs. uncomplicated: 970 (230) μg/L

^a^ PE defined as high blood pressure (≤140/90 mmHg) after 20 weeks gestation and proteinuria (≥300 mg/24 h); ^b^ GH defined as high blood pressure (≤140/90 mmHg) after 20 weeks gestation without proteinuria; ^c^ PE not defined; ^d^ Severe PE not defined; ^e^ Mild PE defined as blood pressure ≥140/90 but less than 160/110 mmHg and severe PE defined as ≥160/110 mmHg; ^f^ PE defined as blood pressure ˃130/85 and proteinuria ≥1 by dipstick, severe PE defined as blood pressure ˃160/110; ^g^ PE defined by the classification proposed by the Paris meeting of the Gestosis Organisation, 1970; ^h^ PE defined as blood pressure ˃ 130/85 and proteinuria ≥1 by dipstick, severe PE defined as blood pressure ˃160/110; ^i^ eclampsia defined as women diagnosed with PE whom also suffer seizures that cannot be attributed to other causes. **Bold print signifies results that were significantly different**. Abbreviations: AAS: atomic absorption spectrometry; GH: gestational hypertension; PE: preeclampsia; SD: standard deviation; SEM: standard error of the mean.

**Table 3:** Included studies assessing maternal zinc status and sPTB.

Author, Country	Sample Size	Zinc Measure	Outcome of the Study
		(1) Sample Type	
		(2) Time at Which Gestation Diet Was Assessed or Sample Collected	
		(3) Method of Analysis	
[44] Scholl, United States ^a^	115 with zinc intake ≤6 mg/day 699 with zinc intake ˃6 mg/day	Dietary zinc intake 28 and 36 weeks 24 h dietary recall	2-fold ↓ risk of delivering a preterm infant with dietary zinc intake ˃6 mg/day. **OR (LMP): 1.85, 95% CI: 1.09–3.12, OR (OE): 2.13, 95% CI: 1.20–3.79** 2.75 to 3.44-fold ↓ risk of delivering a very preterm infant with dietary zinc intake ˃9 mg/day. **OR (LMP): 2.75, 95% Cl: 1.31–5.77, OR (OE): 3.44, 95% Cl: 1.39–8.55**
[94] Carmichael, United States ^a,b^	413 preterm and 58 early preterm 5267 term	Dietary zinc intake Harvard food frequency questionnaires	2-fold ↓ for preterm birth <32 weeks with zinc intake ˃ 8.0 mg/day compared to 8.0–14.2 mg/day. **OR: 2.3, 95% CI: 1.2–4.5**
[45] Neggers, United States ^a^	238 preterm 1160 term	Dietary zinc intake 18 and 30 weeks 24 h dietary recall using the nutrient database developed by the University of Minnesota	NS association between low dietary zinc intake (less than median) and risk of PTB. OR: 1.1, 95% CI: 0.7–1.7
[95] Hsu, Taiwan ^c^	28 preterm 423 term	Dietary zinc intake Each trimester 24 h dietary recall	NS in dietary zinc intake between each of the trimesters and in those who delivered preterm versus term. Preterm: 9.6–10.8 mg/day vs. term: 8.90–10.9 mg/day
*Inadequate dietary zinc intake estimated to affect <17% of the studied population*			
[96] Wang, China ^a^	169 preterm 2912 uncomplicated	Fasting serum zinc First and second trimester Flame AAS	↑ risk of preterm birth with serum zinc <767 μg/L and serum zinc between 767 and 996 μg/L. **aOR: 2.41, 95% CI: 1.57, 3.70; aOR: 1.97, 95% CI: 1.27, 3.05, *p* < 0.001 for both, respectively**
[50] Bro, Denmark ^c^	34 preterm 220 uncomplicated	Serum zinc Collected at delivery Flame AAS	NS mean (SD) serum zinc levels in women who delivered preterm compared to term women. Preterm: 666.7 (104.6) vs. term: 679.7 (98) μg/L
[54] Tamura, United States ^c^	505 preterm and 136 early preterm 2038 uncomplicated	Non-fasting heparin plasma zinc Collected at first prenatal visit (6 to 34 weeks) Flame AAS	NS in the prevalence or n (%) of PTB measured between the lowest quartile and upper three quartiles of zinc. Highest: 373 (14.5) vs. lowest: 132 (15.3) NS in the prevalence (*n* (%)) of early PTB measured between the lowest quartile and upper three quartiles of zinc. Highest: 107 (4.2) vs. lowest: 29 (3.4)
*Inadequate dietary zinc intake estimated to affect ≥17% of the studied population*			
[66] Jeswani, India ^c^	25 preterm 25 term	Serum zinc Collected at 28–40 weeks AAS	↑ mean (SD) serum zinc in women who delivered preterm women compared to term. **Preterm: 1154.4 (154.1) vs. uncomplicated: 962.8 (194.8) µg/L, *p* ˂ 0.01**
[64] Goel, India ^d^	20 preterm 25 term	Heparin plasma zinc Collected at delivery AAS	↑ mean (SD) plasma zinc in mothers who delivered preterm compared to term mothers. **Preterm: 842 (43) vs. term: 744 (51) μg/L, *p* ˂ 0.001**
[60] Bahl, India ^a^	10 preterm 97 term	Serum zinc Collected at delivery Flame AAS	NS mean (SD) in women who delivered Preterm that were an appropriate weight for date compared to uncomplicated. Preterm: 627 (212) vs. uncomplicated: 670 (96) µg/L
[65] Srivastava, India ^c^	26 preterm 23 term	Heparin plasma zinc Collected at delivery Flame AAS	NS mean (SD) plasma zinc between preterm and term mothers. Preterm: 6350 (2640) vs. term: 6310 (5090) μg/L

^a^ PTB defined as ˂37 weeks gestation; ^b^ Early PTB defined as ˂32 weeks gestation; ^c^ PTB defined as ≤37 weeks gestation; ^d^ PTB not defined. **Bold print signifies results that were significantly different**. Abbreviations: AAS: atomic absorption spectrometry; aOR: adjusted odds ratio; CI: confidence interval; LMP: last menstrual period; OE: obstetric estimate; PTB: preterm birth; SD: standard deviation.

**Table 4:** Included studies assessing maternal zinc status and GDM.

Author, Country	Sample Size	Zinc Measure	Outcome of the Study
		(1) Sample Type	
		(2) Time at Which Gestation Diet Was Assessed or Sample Collected	
		(3) Method of Analysis	
[97] Bo, Italy ^a,b^	126 GDM and 84 aOGTT 294 uncomplicated	Dietary zinc intake 24–28 weeks Food frequency questionnaire	↓ mean (SD) daily zinc intake between GDM and aOGTT women and women with uncomplicated pregnancies. **GDM: 8.5 (2.4) and aOGTT: 8.7 (2.5) vs. uncomplicated: 9.4 (2.8) mg/day, *p* = 0.007**
[98] Behboudi-Gandevani S, Iran ^a^	72 with GDM 961 uncomplicated	Dietary zinc intake 14–20 weeks Semi-quantitative food frequency questionnaire	NS in mean (SD) daily zinc intake between GDM and those with uncomplicated pregnancies. GDM: 6.91 (3.42) vs. uncomplicated: 10.1 (7.45) mg/day
*Inadequate dietary zinc intake estimated to affect <17% of the studied population*			
[48] Borella, Italy ^a^	18 GDM 35 uncomplicated	Heparin plasma zinc Collected in the third trimester Flame AAS	↑ mean (SD) plasma zinc in GDM women compared to women with uncomplicated pregnancies. **GDM: 766.6 (117.6) vs. uncomplicated: 627.5 (150) µg/L, *p* ˂0.001**
[35] Wang, China ^a,c^	46 GDM and 98 IGT 90 uncomplicated	Plasma zinc Collection time not specified Inductively coupled plasma atomic emission spectroscopy	NS in mean (SD) plasma zinc between women with IGT and women with uncomplicated pregnancies. IGT: 1080 (270) vs. uncomplicated: 1130 (330) μg/L NS mean (SD) plasma zinc between women with GDM and those with uncomplicated pregnancies. GDM:1020 (190) vs. uncomplicated: 1130 (330) μg/L
[38] Hyvonen-Dabek, Finland ^d^	5 GDM 10 uncomplicated	Serum zinc Collection time not specified Particle induced X-ray emission	NS mean (SD) serum zinc in women with GDM compared to women with uncomplicated pregnancies. GDM: 1070 (190) vs. uncomplicated: 1150 (220) µg/L
[99] Wibell, Sweden ^d^	20 GDM 13 uncomplicated	Serum zinc Collected across gestation AAS	NS mean (SD) serum zinc between women with GDM and those with uncomplicated pregnancies. GDM: 700 (100) vs. uncomplicated: 700 (80) μg/L
*Inadequate dietary zinc intake estimated to affect ≥17% of the studied population*			
[98] Behboudi-Gandevani, Iran ^a^	72 with GDM 961 uncomplicated	Serum zinc Collected 14–20 weeks Flame AAS	NS mean serum zinc between GDM and women with uncomplicated pregnancies. GDM: 844 (440) vs. uncomplicated: 835 (444) μg/L
[100] Al-Saleh, Kuwait ^a^	30 GDM 30 uncomplicated	Serum zinc Collected at delivery Furnace AAS	NS mean (SEM) serum zinc in women with GDM compared to women with uncomplicated pregnancies. GDM: 610.3 (60.1) vs. uncomplicated: 656.2 (241.4) µg/L

^a^ GDM defined as high blood glucose levels in pregnant women who have not previously been diagnosed with diabetes which over a 3 h oral glucose tolerance test provided at least two values over the criteria of Carpenter and Coustan; ^b^ aOGTT defined as high blood glucose levels in pregnant women who have not previously been diagnosed with diabetes which over a 3 h oral glucose tolerance test provided one abnormal value over the criteria of Carpenter and Coustan; ^c^ IGT defined as women with blood glucose consistently higher than 7.8 mmol/L; ^d^ GDM diagnosed with an intravenous glucose tolerance test at 30 weeks gestation. **Bold print signifies results that were significantly different**. Abbreviations AAS: atomic absorption spectrometry; aOGTT: abnormal oral glucose tolerance test; BMI: body mass index; GDM: gestational diabetes mellitus; IGT: impaired glucose tolerance; OGTT: oral glucose tolerance test; SD: standard deviation.

**Table 5:** Summary of all the studies reviewed and whether zinc status was positively, negatively or not associated with the studied pregnancy complication.

**Dietary Zinc Intake**				
Total No. Reference	LBW/SGA	Hypertensive Disorders of Pregnancy	sPTB	GDM
9	4	1	4	2
	3 reported a negative association [42,43,44]	Reported no association [70]	2 reported a negative association [44,93]	1 reported a negative association [96]
	1 reported no association [45]		2 reported no association [45,94]	1 reported no association [97]
**Serum/Plasma Zinc**				
Total No. Reference	LBW/SGA	Hypertensive Disorders of Pregnancy	sPTB	GDM
58	26	33	7	6
*No. where inadequate zinc intake affects <17% of the population*				
	12	13	3	4
	2 reported a negative association [46,49]	5 reported a negative association [55,71,72,73,74]	1 reported a positive association [95]	1 reported a positive association [48]
	2 reported a positive association [47,48]	8 reported no association [38,48,53,75,76,77,78,79]	2 reported no association [50,53]	3 reported no association [35,38,98]
	8 reported no association [38,50,51,52,53,54,55,56]			
*No. where inadequate zinc intake affects ≥17% of the population*				
	14	20	4	2
	5 reported a negative association [57,58,59,60,61]	10 reported a negative association [33,34,36,37,39,80,81,82,83,100]	2 reported a positive association [64,66]	2 reported no association [97,99]
	2 reported a positive association [63,101]	1 reported a positive association [84]	2 reported no association [60,65]	
	6 reported no association [64,65,66,67,68,69]	9 reported no association [32,85,86,87,88,89,90,91,92]		

Abbreviations: GDM: gestational diabetes mellitus; LBW: low birth weight; SGA: small for gestational age; sPTB: spontaneous preterm birth.

## Appendix A

Outline of the search terms and MeSH headings identified in the Medline search and used for the remaining database searches.

**Table:** Search strategy: MEDLINE (OVID).

	Searches	Results
1	exp Zinc/ or zinc.mp.	108,695
2	plasma zinc.mp.	1365
3	zinc intake.mp.	679
4	dietary zinc.mp.	1158
5	serum zinc.mp.	2083
6	1 or 2 or 3 or 4 or 5	108,695
7	preterm birth.mp. or exp Premature Birth/	14,642
8	premature birth.mp. or Premature Birth/	11,131
9	small for gestational age.mp.	9255
10	exp Infant, Small for Gestational Age/	5977
11	gestational hypertension.mp. or exp Hypertension, Pregnancy-Induced/	32,078
12	pre?eclampsia.mp. or exp Pre-Eclampsia/	30,863
13	exp Pre-Eclampsia/ or exp Eclampsia/ or eclampsia.mp.	32,471
14	exp HELLP Syndrome/ or HELPP syndrome.mp.	1613
15	gestational diabetes.mp. or exp Diabetes, Gestational/	11,382
16	fetal macrosomia.mp. or exp Fetal Macrosomia/	2369
17	7 or 8 or 9 or 10 or 11 or 12 or 13 or 14 or 15 or 16	69,148
18	infant, low birth weight.mp. or Infant, Low Birth Weight/	16,571
19	infant, very low birth weight.mp. or exp Infant, Very Low Birth Weight/	8491
20	17 or 18 or 19	89,771
21	6 and 20	380
22	limit 21 to (english language and full text and humans)	165

## Appendix B

**Table:** List of conversion factors used to convert all measures of zinc to μg/L.

Units	Conversion
μg/100 mL or μg/dL	Multiply 10
μmol/L or μM	Divide 0.153
mg/L	Multiply 1000
μg/mL	Multiply 1000
